# Catheter Ablation for Atrial Fibrillation in Patients with Hemophilia or von Willebrand Disease

**DOI:** 10.1055/s-0039-1698756

**Published:** 2019-10-24

**Authors:** Paul R. van der Valk, Eveline P. Mauser-Bunschoten, Jeroen F. van der Heijden, Roger E. G. Schutgens

**Affiliations:** 1 Van Creveldkliniek, University Medical Center, University Utrecht, Utrecht, The Netherlands; 2 Division Heart and Lung, Cardiology, Electrophysiology, UMC Utrecht, Utrecht, The Netherlands

**Keywords:** atrial fibrillation, Hemophilia A, von Willebrand Disease, catheter ablation, anticoagulant, bleeding

## Abstract

**Background** Management of atrial fibrillation (AF) is complex in patients with bleeding disorders. Catheter ablation such as pulmonary vein isolation (PVI) has been suggested in cases with bleeding disorders. However, data on safety are missing. This report describes the outcome of PVI in patients with bleeding disorders.

**Methods** A retrospective study in our hemophilia treatment center of patients who underwent a PVI in 2014 to 2018. PVI was done according to local protocol. Clotting factor was given periprocedural. Postprocedural anticoagulation was given for at least 4 weeks, with clotting factor suppletion if needed to maintain factor VIII (FVIII) levels >0.20 IU/mL.

**Results and Discussion** Five patients with hemophilia and one with von Willebrand disease were included. Eight PVIs were performed. Target FVIII levels (>0.80 IU/mL) were met before the procedure. Postprocedural anticoagulation was given: vitamin K antagonist (VKA) or direct oral anticoagulant (DOAC) dabigatran. All patients obtained long-term sinus rhythm, in two patients after a second PVI. However, late recurrent AF occurred in one patient after 42 months. A notable incidence of groin bleeds was observed: two of eight interventions (25%) compared with 0.9% in the general population. Bleeding seemed to be related to agitation, early mobilization, and bridging of VKA with low molecular weight heparin (LMWH). No relevant bleeding was observed when on DOAC therapy.

**Conclusion** PVI seems to be effective in the case of bleeding disorders. To reduce the groin bleeds agitation and early mobilization should be avoided and DOAC is preferred over bridging VKA with LMWH.

## Introduction

Life expectancy in patients with hemophilia (PWHs) has increased significantly to almost normal. 1 They are therefore more likely to be confronted with age-related comorbidity like cardiovascular disease (CVD). CVD mortality has been reported to be lower than the general population but numbers are increasing. 2 3 A common CVD in the aging population is atrial fibrillation (AF). AF can cause ischemic stroke. The risk of an ischemic stroke in patients without bleeding disorders can be estimated with the so-called CHA _2_ DS _2_ -VASC score, 4 but its applicability in hemophilia is questioned. 5 Management of PWH and AF is complex in finding the balance between coagulation and anticoagulation. In a large cross-sectional study, the prevalence of AF in PWH was comparable with that of the general population; however, anticoagulation management was diverse. 6 The most challenging patients are those with a high bleeding and stroke risk. In these patients, we question if the use of catheter ablation (like pulmonary vein isolation [PVI]) is a feasible option to treat these patients. However, the feasibility of PVI in patients with bleeding is unknown. Here we describe our experience in patients with bleeding disorders undergoing PVI to treat their arrhythmia-related symptoms.

## Methods

In this retrospective single-center cohort, we collected data from patients visiting the hemophilia treatment center (Van Creveldkliniek, University Medical Center Utrecht, The Netherlands) who underwent a PVI between 2014 and 2018. Data on baseline characteristics, type of bleeding disorder, comorbidity, reason for PVI, and procedural outcomes were extracted from the medical files. This project was approved by the local medical ethical committee.

### Pulmonary Vein Isolation

Prior to PVI, evaluation of three-dimensional (3D) cardiac imaging was done by a cardiac computed tomography (CT) scan providing detailed left atrial anatomy and confirming the absence of a left atrium appendage thrombus.

Electrophysiological studies were performed in a sedated, fasting state. Catheters were introduced through the right femoral vein using three sheaths (once 7 French and twice 8.5 French). An octapolar catheter was positioned in the coronary sinus. The left atrium was reached by means of a transseptal puncture. A 3D cardiac mapping system (Carto; Biosense Webster, Diamond Bar, California, United States) was used to obtain a 3D reconstruction of the cardiac anatomy. An irrigated tip catheter (ThermoCool SmartTouch, Biosense Webster) was used to widely encircle the right and left pulmonary veins (PVs) at their antrum with point-by-point ablation lesions. Procedures were performed as described before. 7 Endpoint of ablation procedure was electrical isolation of all PVs, as determined with the circular mapping catheter. PV isolation had to be consistent after a waiting period of 30 minutes after the last ablation application.

At the end of the procedure, sheaths were removed followed by manual compression of the femoral vein for at least 10 minutes. Thereafter compression dressing was placed for a least 4 hours and after its removal additional bedrest was continued for another 4 hours.

### Definition of Success

There were two different definitions of success. During the “blanking period,” the period after the procedure, reoccurrence of AF is normally not seen as failure.

Success rate of single PVI: no signs of AF following the 3-month “blanking period” through the last follow-up. Overall success rate: no AF following one or more PVIs and a 3-month “blanking period” through the last follow-up. 8

### Coagulation Management

Patients with baseline FVIII > 0.20 IU/mL started at least 4 weeks prior to PVI with anticoagulation: vitamin K antagonist (VKA) or direct oral anticoagulant (DOAC) dabigatran 110 mg twice daily.

Clotting factor correction aiming at a peak FVIII level of 0.8 to 1.0 IU/mL was started 1 hour before the procedure. Target trough levels were aimed at 0.5 IU/mL and peak levels were maintained at 0.8 to 1.0 IU/mL for the first 24 hours, for which two additional bolus injections were given with 12-hour interval.

Anticoagulation during the PVI procedure was according to the local standard protocol with unfractionated heparin (UFH) therapy with a target activated clotting time (ACT) of 300 to 350 seconds.

Before April 2016, 4 to 8 hours after the procedure therapeutic dosed low molecular weight heparin (LMWH: dalteparin, twice daily 100 IE/kg) was started in combination with VKA. LMWH was stopped when a therapeutic international normalized ratio (INR) > 2.0 was obtained. Duration of VKA therapy was dependent on the extent and result of PVI, with a minimum of 4 weeks. During VKA therapy FVIII trough levels were kept above 0.2 IU/mL.

Since April 2016 dabigatran (110 mg twice-daily) was used instead of VKA therapy, avoiding the need for bridging with LMWH during PVI. In patients with FVIII > 0.2 IU/mL, it was started 4 weeks before the procedure. In patients with FVIII < 0.2 IU/mL, it was started 4 to 8 hours after the procedure. Postprocedural DOAC was continued for at least 4 weeks, in combination with FVIII suppletion with a target FVIII trough level above 0.2 IU/mL.

## Results

### Patients

Five patients with hemophilia A and one with von Willebrand disease underwent a total of eight PVIs at a mean age of 62.2 years ( Table 1 ). Two patients had severe hemophilia and three mild. One patient had a history of a past inhibitor against FVIII, but currently has a normal FVIII half-life. Median duration of AF before the first PVI was 5 (interquartile range [IQR]: 4.75–7) years. Median CHA _2_ DS _2_ -VASC score was 0.5 (IQR: 0–2.25). All patients were suffering from symptomatic AF. Median follow-up after the last PVI was 33 months (IQR: 10.25–43.75).

**Table 1:** Patient characteristics

Patient	Age	Bleeding disorder	Clotting factor level (IU/mL)	Reason for intervention	CHA _2_ DS _2_ VASC	Duration (y)	Prior therapy	On chronic anticoagulation before PVI
1	70	HA	FVIII 0.35	AF: dyspnea and fatigue	3	5	CV, ECV	VKA
2	72	HA	FVIII < 0.01	pAF: with severe fatigue	1	7	BB	No
3	59	HA	FVIII 0.23	pAF: bradycardia with decreased ejection fraction	0	4	ECV, flecainide	No
4	50	HA	FVIII < 0.01	pAF: frequent tachycardia	0	5	CV, flecainide, BB	No
5	55	HA	FVIII 0.06	pAF: persistent paroxysm under medication	0	5	Flecainide, BB	No
6	67	VWD	vWF RCo 19% FVIII 0.50	AF: dyspnea and fatigue	2	7	VATS Maze; ECV	No

Abbreviations: AF, atrial fibrillation; BB, beta-blocker; CV, chemical cardioversion; ECV, electric cardioversion; HA, hemophilia A; pAF, paroxysmal atrial fibrillation; RCo, ristocetin cofactor (IU/dL); VWD, von Willebrand disease; vWF, von Willebrand factor; VATS Maze: video assisted thoracoscopic surgery.

**Table 2:** Interventions—outcome and complications

Intervention		Patient	Outcome	AC before	AC after procedure	Stopped VKA/DOAC	Periprocedural groin bleeding
1	PVI	1	SR, later pAF	VKA	3 mo VKA (LMWH b )	No: recurrent pAF	Day 5: Hb drop 3.22 g/dL
2	PVI (redo)	1	SR	Dabigatran a	Dabigatran a	No	No
3	PVI	2	SR, later pAF	No	1 mo VKA (LMWH b )	Yes, as planned after 1 mo	Day 3: Hb drop 4.83 g/dL
4	PVI (redo)	2	SR under sotalol	No	1 mo VKA (LMWH b ), 2 mo ASA	Yes, as planned after 1 mo	Postprocedure oozing during 4 h
5	PVI	3	SR	Dabigatran a	6 mo dabigatran a	Yes, after 6 mo	No
6	PVI	4	SR	No	6 wk VKA (LMWH b )	Yes	No
7	PVI	5	SR	No	6 wk dabiagtran a	Yes	No
8	PVI	6	SR	No	6 wk dabigatran a	Yes	No

Abbreviations: AC, anticoagulation; ASA, 38 mg acetylsalicylic acid; Hb, hemoglobin: 3.22 g/dL = 2.0 mmol/L; 4.83 g/dL = 3.0 mmol/L; pAF, paroxysmal atrial fibrillation; PVI, pulmonary vein isolation; SR, sinus rhythm; VKA, vitamin K antagonist: target INR 2.0–3.0.

a 110 mg BID 4 weeks before PVI, last dose 24 h before intervention.

b Therapeutic LMWH until therapeutic INR.

### Before PVI

Two patients were on anticoagulation therapy before the PVI ( Table 2 ). Patient 1 (with a CHA _2_ DS _2_ -VASC 3 and FVIII 0.35 IU/mL) was on long-term VKA therapy before his first PVI and on long-term dabigatran before the second PVI. The other (patient 3) was on dabigatran before PVI for 4 weeks.

### During PVI

According to the protocol, during all the procedures, boluses of UFH were administered, based on the ACT results, without any complications.

In all cases of hemophilia, target FVIII levels periprocedural were met. The mean dose of the first bolus was 31.7 IU/kg (IQR: 16.9–43.9) and the mean peak FVIII was 1.14 IU/mL. The patient with von Willebrand disease reached a peak von Willebrand factor activity level of 0.66 IU/mL, despite weight-based suppletion of 3,000 IU of Von Willebrand factor (Wilfactin; LFB, Les Ulis, France) half an hour before the blood collection. The FVIII level was at that moment 0.52 IU/mL).

### After PVI

Several hours (4–8 hours) after the procedure, therapeutic LMWH and VKA ( *n* = 4) or dabigatran ( *n* = 4) were started.

For anticoagulation management after PVI, see Table 2 . VKA anticoagulation was stopped in three out of four procedures after a mean of 4.7 weeks. Dabigatran was stopped in three out of four procedures after a mean use of 12.7 weeks. Patient 1 continued AC after both procedures because of a CHA _2_ DS _2_ -VASC 3 and factor level > 0.20 IU/mL.

Four interventions were followed by intensive factor suppletion during 4 to 6 weeks (mean duration of 5 weeks) aiming and achieving trough FVIII levels of > 0.20 IU/mL. The mean total clotting factor use was 76,625 IU (824 IU/kg) during this period.

### Success rates

The success rate of a single PVI was 67% (4/6); for two recurrent AFs, a second PVI (9 and 16 months after the first PVI) was performed ( Table 2 ).

Only two patients met the definition of long-term follow-up of over 36 months, with a 100% success rate. However, the final overall success rate was five out of six: one patient had recurrent AF 42 months after the second PVI.

### Complications

Clinically relevant bleeding complications (grade 2 World Health Organization bleeding scale) occurred from the femoral vein puncture in two out of eight procedures ( Table 2 ).

Patient 1 developed a small groin hematoma on the day of the procedure. He was agitated during the procedure and nonadherent to bedrest. His FVIII level was completely corrected for 36 hours. He was on therapeutic LMWH therapy before the procedure because of INR of <2.0 despite VKA. LMWH was stopped the evening after the procedure, because an adequate INR of 3.2 was obtained. He was discharged with a stable hematoma; hemoglobin level was not measured. Five days later he was readmitted in another hospital with a progressive groin hematoma and low hemoglobin (−5.16 g/dL) and a FVIII of 0.71 IU/mL. His INR at admission was 2.4. However, he had a good periprocedural FVIII recovery and trough levels and no inhibitor; it was assumed that his FVIII levels after discharge from, and at readmission at, the hospital must have been at least 0.35 IU/mL. He was successfully treated with high-dose clotting factor concentrate for 1 day to stop bleeding. The hematoma completely resolved, and patient functionally fully recovered.

In patient 2 the procedure was complicated by an acute coughing spell. He reported a progressive discomfort and hematoma of his groin 3 days after the procedure with decreased hemoglobin. At that time his trough FVIII level was 0.31 IU/mL (the lowest during his hospital stay), the INR was 3.0, and he was still on therapeutic LMWH therapy. Because of the bleeding, FVIII was continued at higher dose aiming at trough levels of >0.40 IU/mL and LMWH was stopped. He was discharged with 2 days delay. VKA was continued and FVIII was given (1,500 IU once-daily) aiming at trough levels >0.40 IU/mL for 2 weeks. The patient fully recovered within these 2 weeks and thereafter the target through level was decreased to 0.20 IU/mL.

Both patients with a severe groin bleed needed a redo PVI because of recurrent AF. Patient 1 was sedated during the second procedure and additional clotting factor correction was given on day 4, to prevent late (re)bleeding. This time he was treated with dabigatran, a DOAC, instead of VKA. No bleeding occurred. The redo PVI in patient 2 was complicated by mild oozing from the puncture site for several hours while FVIII levels were corrected completely.

None of the patients developed thrombocytopenia or other bleeding tendency.

## Discussion

In this small cohort study the success rate of PVI seems comparable with that of the general population. The success rate of a single PVI was 67% and after a second PVI 83% for achieving sinus rhythm. In the literature a success rate of up to 70% of patients with paroxysmal AF, and around 50% in persistent AF is found, with most patients requiring more than one procedure. 4

In the general population 5 to 7% of patients suffer from severe complications after PVI, of which 2 to 3% are life-threatening, but usually manageable. The most severe complications are stroke (<1%) and pericardial tamponade (1–2%). 4 The incidence of clinical relevant groin hematoma after PVI in the general population is 0.9%. 9 In our group it is likely that the combination of therapeutic LMWH, VKA therapy, decreased FVIII levels and mobilization shortly after the procedure have caused the high bleeding rate (25%). In an observational study in the general population, bridging during ablation was associated with more bleeding complications, compared with continuing VKA. 10 Bridging was also associated with an increased stroke risk. 11 DOAC, however, is associated with a low incidence of stroke or transient ischemic attack and a significant reduction of major bleeding. 12 In this small cohort we see the historical transition from bridging to nonbridging with a DOAC; in this small group no bleeding under DOAC was seen. We have chosen the DOAC dabigatran, a direct thrombin inhibitor, in a lower dose (110 mg twice daily) for patients with a bleeding disorder and an indication for anticoagulation. With this DOAC we have the option to reverse the anticoagulant if needed with idarucizumab. 13 Furthermore, FVIII levels can be measured in case of emergency, in contrast to oral anti-Xa inhibitors that interfere with our laboratory assays.

Applying anticoagulation therapy in patients with bleeding disorders is complex.

AF has the risk of atrial thrombus embolization and ischemic stroke. The applicability of the CHA _2_ DS _2_ -VASC score 4 in patients with clotting factor deficiency is questioned. 5 There is lack of data on stroke and bleeding risk during anticoagulation in a population with increased bleeding tendency. As there are no prospective trials, a European working group (ADVANCE) gathered consensus for the management of AF in PWHs. 5 Recently we reported an updated approach in anticoagulation management in PWH with AF. 14 We suggest to assess the bleeding risk based on clotting factor level and the thrombotic risk using the CHA _2_ DS _2_ -VASc score using adjusted cutoffs. A factor level of 0.20 IU/mL is thought to be safe for treatment with anticoagulation.

There is no data for the periprocedural management of coagulation for PVI.

Normally patients not on anticoagulation start 4 weeks before the PVI with anticoagulation. According to our protocol only patients with factor levels above 0.20 IU/mL were treated this way. Both patients did not report bleeding complications. On the other hand, patients not on anticoagulation did not have embolic events during this short time. For the future we are considering to withhold anticoagulation in patients with a low CHA _2_ DS _2_ -VASc score (<4) with confirmation of absence of left atrium appendage thrombus by cardiac CT-scan before the procedure.

During PVI clotting factor suppletion is necessary to prevent a groin bleed and therefore anticoagulation is needed.

Following PVI, patients are treated with anticoagulation and in the case of FVIII levels <0.20 IU/mL, daily factor suppletion for at least 4 weeks. At this moment we think that withholding anticoagulation in patients with FVIII <0.20I U/mL is not possible, because of an expected high risk of thrombus formation on the large coagulation area in the cardiac atria.

In our cohort clinically relevant bleeding occurred 3 to 5 days after the procedure, a delay not uncommon in hemophilia as clot formation is impaired. Treatment consisted of stopping LMWH, which was already planned because of adequate INR, and increasing the FVIII target level for a limited time. We choose not to antagonize VKA, because postprocedural patients needed to be anticoagulated with VKA for a longer time.

## Conclusion

Our results suggest that PVI is effective and a generally safe procedure in patients with bleeding disorders, provided that a strict control of hemostasis is attained and preferably a DOAC is used as anticoagulation. Because of the unexpected significant groin hematomas in two patients (on VKA with LMWH bridging) despite complete clotting factor correction for at least 24 hours, agitation during and the first hours after the procedure and early mobilization should be avoided. Furthermore, also in patients with bleeding disorders, DOAC seems safer compared with bridging of VKA with LMWH.
